# Dysregulation of Hedonic Processing in Chronic Pain: Insights from Preclinical Data

**DOI:** 10.3390/brainsci15121265

**Published:** 2025-11-25

**Authors:** Mariana Cerqueira-Nunes, Clara Monteiro, Vasco Galhardo, Helder Cardoso-Cruz

**Affiliations:** 1 Instituto de Investigação e Inovação em Saúde, Pain Neurobiology Group, Universidade do Porto, Rua Alfredo Allen 208, 4200-135 Porto, Portugal; mariana.nunes@i3s.up.pt (M.C.-N.); cmonteir@med.up.pt (C.M.); galhardo@med.up.pt (V.G.); 2 Instituto de Biologia Molecular e Celular, Universidade do Porto, Rua Alfredo Allen 208, 4200-135 Porto, Portugal; 3 Faculdade de Medicina, Departamento de Biomedicina—Unidade de Biologia Experimental, Universidade do Porto, Rua Doutor Plácido da Costa, 4200-450 Porto, Portugal; 4 Programa Doutoral em Neurociências, Faculdade de Medicina, Universidade do Porto, Rua Doutor Plácido da Costa, 4200-450 Porto, Portugal

**Keywords:** chronic pain, hedonic processing, anhedonia, motivational control, dopamine, cognitive dysfunction

## Abstract

Chronic pain has been increasingly recognized not only as a sensory disorder but also as a condition that profoundly disrupts affective and motivational processes. Preclinical research indicates that persistent nociceptive input drives maladaptive changes in brain reward circuits, particularly within the mesocorticolimbic dopamine system. These alterations contribute to anhedonia, diminished motivation, and aberrant reward valuation, core symptoms frequently observed in patients with chronic pain. In this review, we synthesize evidence from rodent models demonstrating how chronic pain impairs the hedonic experience and motivational aspects of reward through disrupted dopaminergic signaling, neuroinflammatory pathways, and opioid system dysregulation. We also highlight the temporal dynamics in the emergence of hedonic deficits, the sex-dependent mechanisms, and the interplay between pain and drug-seeking behaviors. Finally, we discuss how these findings inform the search for and predict early biomarkers and therapeutic targets aimed at restoring hedonic tone. A deeper understanding of the neurobiological basis of reward dysfunction in chronic pain may provide critical insights for developing more effective, mechanism-based interventions.

## 1. Introduction

Pain is a multifaceted experience encompassing sensory, cognitive, and motivational dimensions [1]. Acute pain serves an adaptive role, signaling tissue damage and promoting avoidance behaviors to facilitate healing. In contrast, chronic pain—defined as persistent pain lasting beyond three months or expected tissue recovery—loses its protective function, becoming a maladaptive central nervous system condition [2,3,4]. This transition induces widespread neural plasticity, particularly in circuits controlling mood, cognition, and reward, transforming pain from a symptom into a disease state [5,6]. In this regard, chronic pain is strongly associated with psychiatric comorbidities, including depression, anxiety, and cognitive impairments [7,8,9]. A hallmark feature is anhedonia (the reduced ability to experience pleasure), which is prevalent yet often underdiagnosed in chronic pain patients. As a core symptom of major depressive disorder, it reflects a distinct neuropsychiatric overlap with chronic pain, contributing to reduced quality of life, social withdrawal, and increased suicide risk [10,11,12]. Preclinical models of neuropathic and inflammatory pain in rodents consistently replicate these anhedonic behaviors, offering insights into underlying mechanisms [13,14]. Moreover, neuroimaging and electrophysiological studies reveal that chronic pain disrupts reward-processing circuits, including the nucleus accumbens (NAc), ventral tegmental area (VTA), prefrontal cortex (PFC), orbitofrontal cortex (OFC), and amygdala, which form the mesocorticolimbic system [15,16,17,18,19,20]. Dopaminergic (DAergic) projections from the VTA to the NAc mediate motivational salience, while opioidergic and endocannabinoid signaling in the NAc shell area (NAcSh) and ventral pallidum (VP) regulate hedonic experience [21,22,23]. Chronic pain impairs these interactions, leading to diminished motivation and reward responsiveness. Recent works demonstrate that rodents with neuropathic or inflammatory pain exhibit reduced reward-seeking and hedonic responses, accompanied by DAergic hypofunction, neuroinflammatory changes, and opioid system dysregulation in mesolimbic pathways [13,14,24,25,26]. These deficits may vary with sex, with females often showing greater affective impairments due to specific hormonal influence [27,28]. In part, these alterations explain why analgesics targeting nociceptive pathways, such as nonsteroidal anti-inflammatory drugs or opioids, often fail to address affective and motivational deficits. However, emerging evidence suggests that interventions like D2 receptor agonists, κ-opioid receptor (KOR) antagonists, or deep brain stimulation may restore hedonic tone and reward function [26,29,30,31,32].

**Figure 1:**
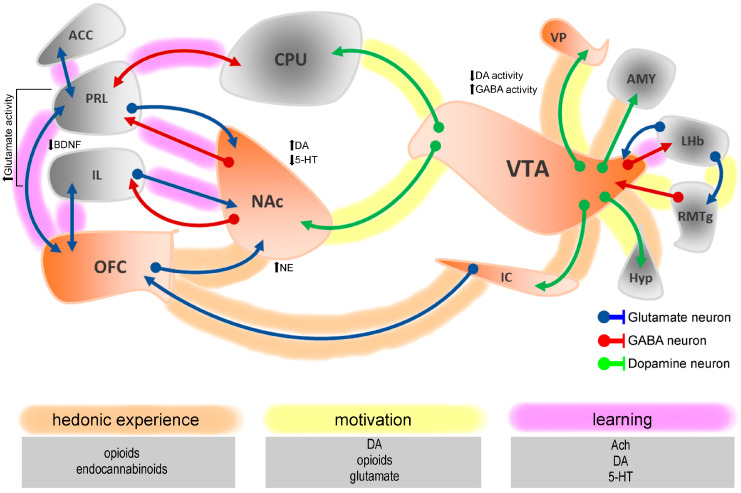
Simplified conceptual model of intra-hemispheric hedonic circuit inputs and outputs with pain-related effects on their respective neurotransmitter systems. Liking hedonic hotspots are indicated in orange, and wanting hotspots are indicated in gray. Abbreviations: 5-HT, serotonin; ACC, anterior cingulate cortex; Ach, acetylcholine; AMY, amygdala; BDNF, brain-derived neurotrophic factor; CPU, caudate nucleus and putamen; DA, dopamine; GABA, gamma-aminobutyric acid; Hyp, hypothalamus; IC, insular cortex; IL, infralimbic cortex; LHb, lateral habenula; NAc, nucleus accumbens; NE, norepinephrine; OFC, orbitofrontal cortex; PRL, prelimbic cortex; RMTg, rostromedial tegmental nucleus; VP, ventral pallidum; VTA, ventral tegmental area.

Hedonic processing relies on an interconnected neural network, primarily the mesocorticolimbic reward system. The NAcSh, a hedonic hotspot, is modulated by μ-opioid receptors (MORs) and endocannabinoid signaling, amplifying positive affective reactions to rewards like dulcets [21,33]. The VP further enhances these hedonic responses through the same mechanisms. Conversely, DAergic projections from the VTA to the NAc core (NAcC), PFC, and OFC drive motivational salience, determining effort for rewards without altering hedonic impact [22,34]. Specifically, the medial OFC encodes subjective reward value by integrating multisensory and physiological inputs [35,36]. These regions operate in dynamic loops, such as the VTA–NAc–OFC circuit, which permit the seamless integration of sensory, motivational, and cognitive information essential for the hedonic experience (Figure 1). Chronic pain disrupts these circuits, reducing NAc DAergic tone and increasing medial PFC inhibitory input to VTA neurons, promoting avoidance and motivational anhedonia [32,37,38]. In the case of neuropathic pain, reduced NAc dopamine (DA) release correlates with anhedonia, reinforcing these circuits’ role in chronic pain [13]. Chemogenetic VTA DA activation increases sucrose-seeking effort in rats, suggesting a strategy to counter motivational deficits [39]. These disruptions may be sex-specific, with females showing greater DAergic impairments due to estrogen modulation, ultimately exacerbating motivational deficits [40]. Estrogen modulates DAergic signaling by enhancing DA synthesis and turnover and regulating D1/D2 receptor expression in the PFC and striatum. These regions are central to executive function, motivation, and reward processing [41]. Moreover, membrane-associated estrogen receptors located on DAergic neurons further influence receptor expression and sensitivity [41,42,43]. Collectively, these mechanisms contribute to sex differences in DA-dependent motivation and cognition, highlighting estrogen’s key role in frontostriatal modulation during chronic pain. Beyond the mesocorticolimbic core, other regions modulate hedonic processing in chronic pain. The anterior cingulate cortex (ACC) resolves motivational conflicts [44,45], the anterior insula shapes affective awareness [46], and the amygdala, with its hippocampal input, processes emotional salience and reward memory [47,48,49]. This dissociation underscores the need for circuit-specific therapies, such as KOR antagonists or DA agonists, to address motivational deficits in chronic pain while preserving hedonic capacity [29]. By elucidating the neurobiological basis of reward dysfunction in chronic pain, preclinical research paves the way for mechanism-based therapies that target both sensory and affective dimensions of the disorder. Clinical evidence aligns with this translational perspective. For instance, a recent study of 174 patients with neuropathic pain reported no sex differences in the efficacy or safety of multimodal treatments, despite females exhibiting higher rates of anxiety and depression [50]. This dissociation between treatment response and affective burden mirrors the sexually dimorphic hedonic impairments described in preclinical models and underscores the need for reward-focused, sex-informed therapeutic strategies.

## 2. Neural Basis of Pain-Induced Neurochemical and Neuroanatomical Changes in Hedonic Processing

**Figure 2:**
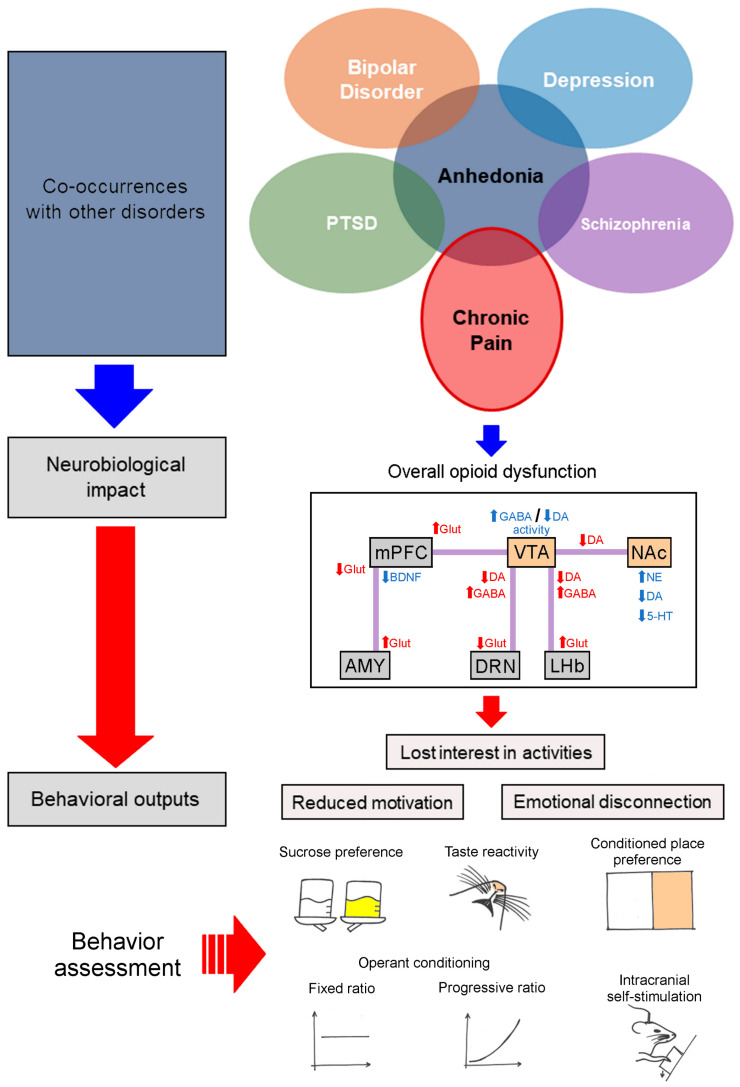
Diagram illustrating the relationship between chronic pain, anhedonia, and co-occurring neuropsychiatric disorders. Chronic pain and anhedonia share overlapping features that are commonly observed across bipolar disorder, depression, schizophrenia, and post-traumatic stress disorder (PTSD). These comorbidities exert neurobiological impacts, including decreased DAergic activity, increased inhibitory signaling, and glutamatergic disruptions contributing to impaired behavioral outcomes. The bottom section illustrates how these neurobiological alterations translate into measurable deficits in hedonic processing. Chronic pain–related dysfunction across key motivational and reward-related regions manifests as reduced motivation, emotional disconnection, and loss of interest in rewarding activities. These changes are assessed through established behavioral assays, including sucrose preference, taste reactivity, conditioned place preference, operant conditioning (fixed and progressive ratio schedules), and intracranial self-stimulation, each providing quantifiable indicators of pain-related disruptions in reward function. Abbreviations: AMY, amygdala; BDNF, brain-derived neurotrophic factor; GABA, gamma-aminobutyric acid; Glut, glutamate; DA, dopamine; DRN, dorsal raphe nucleus; LHb, lateral habenula; mPFC, medial prefrontal cortex; NAc, nucleus accumbens; VTA, ventral tegmental area.

Hedonic processing involves a complex distributed network of brain regions shared across rodents and humans [21,51]. These hedonic hotspots mediate conscious experience of pleasure and how positive affect or unconscious “liking” is generated. Conversely, incentive salience or “wanting” describes how stimuli become attractive and motivationally significant. The “wanting” network is highly influenced by DA and can embed this component in environmental cues to drive the pursuit of goals and rewards. Dysfunction in one or more components of reward, either hedonic impact (“liking”), motivational salience (“wanting”), or learning (associations and predictions), can lead to anhedonia. In this context, chronic pain triggers profound neurochemical and anatomical remodeling in the brain regions that govern reward processing, particularly within the mesolimbic DA system (Figure 2). This system includes the NAc, VTA, medial PFC, and amygdala, and is essential for mediating both the hedonic and motivational aspects of reward. In this regard, a study by de la Puente et al. [13], using a partial sciatic nerve ligation (PSNL) model in mice, provided compelling evidence of chronic pain-induced disruptions in this network. Specifically, mice with neuropathic pain showed significantly reduced extracellular DA concentrations in the NAc along with increased norepinephrine (NE) levels. In this context, microdialysis and HPLC (high-performance liquid chromatography) analyses revealed blunted DAergic and serotonergic (5-HTergic) responses to palatable stimuli, despite normal motor function. Behaviorally, the mice exhibited reduced sucrose preference and decreased operant responding for food rewards, both indicators of anhedonia. Furthermore, the study identified the σ-1 receptor as a modulator of the disrupted reward signaling. Pharmacological blockade of this receptor using the E-52862 ligand restored DA and serotonin (5-HT) levels in the NAc and reversed motivational deficits, suggesting a promising target for pain-induced mood disorders [13]. Adding to this, pharmacological manipulations of the NAc have been shown to increase the hedonic impact of food, as measured by facial reactivity [51] and number of licks per bout of consumption [52,53]. Drugs that promote positive taste reactions are generally inhibitory, including opioids [53,54], cannabinoids [55], and gamma-aminobutyric acid receptor (GABA) agonists [56]; however, their influences on NAc activity are complex since they differentially modulate NAc cell types and afferent pathways [57,58]. For example, reduced activity in D1 receptor-expressing neurons in the NAcSh that project to the lateral hypothalamus has been shown to be both necessary and sufficient to drive food consumption [59]. However, whether this modulation also alters the hedonic value of the consumed stimuli remains unclear. It is therefore equally plausible that reduced activity of D2 receptor-expressing neurons may enhance positive affect, as their activation is often associated with aversive states and depressive-like behaviors [60,61]. Adding to this, Pekarskaya et al. [62] show that disrupting MOR signaling in the habenula exacerbates negative affect and increases nociceptive sensitivity. These findings provide novel mechanistic insight into how opioid dysfunction within the epithalamic region contributes to the emotional burden of pain, offering some of the strongest evidence to date that the habenula orchestrates affective behaviors associated with nociception. Complementing this, Liu et al. [63] reveal that chronic pain induces divergent adaptations within discrete NAc pathways, with some circuits driving nociceptive amplification and others mediating depression-like states. Together, these studies highlight how circuit-specific changes in the NAc produce parallel sensory and affective disturbances, underscoring its central role in pain-related mood disorders. At a molecular level, Guimarães et al. [64] reveal that chronic pain–induced anhedonia is associated with disruptions in the accumbal ubiquitin–proteasome system, indicating that cellular proteostasis mechanisms contribute to reward deficits beyond circuit-level adaptations. Extending this perspective, Serafini et al. [65] demonstrate that persistent neuropathic pain is maintained in part through dysregulated transcriptional signaling within the NAc via myocyte enhancer factor 2C (MEF2C). Targeted manipulation of MEF2C alleviates negative affect and anxiety-like behaviors while restoring dopaminergic neurotransmission, establishing MEF2C as a key molecular regulator linking long-term nerve injury to maladaptive reward circuitry and affective dysfunction.

Beyond the NAc, chronic pain also disrupts DAergic signaling in the VTA, the origin of mesolimbic DA neurons. Chronic nociceptive input has been shown to increase GABAergic inhibition of VTA DA neurons, particularly through augmented excitatory glutamatergic input from the mPFC onto VTA GABA interneurons [66]. This results in a sustained reduction in the excitability of DA neurons and diminished phasic DA release in downstream targets, including the NAc. Importantly, optogenetic activation of VTA DA neurons has also been shown to rescue these deficits in rats. For example, Markovic et al. [32] demonstrated that light-induced stimulation of VTA DA neurons in spared nerve injury (SNI) mice restored operant performance for sucrose rewards to baseline levels, directly implicating reduced DAergic tone as a causal factor in pain-induced anhedonia. Additional studies support the idea that inhibitory control within the VTA is a crucial modulator of reward behavior. Van Zessen et al. [67] showed that optogenetic stimulation of VTA GABAergic neurons following reward delivery suppressed reward consumption by directly inhibiting neighboring DA neurons, therefore reducing DA release in the NAc. Furthermore, Zhu et al. [68] recently reported that VTA DA neuron activity increases during food consumption in a phase-specific manner in rodents. They identified peri-locus coeruleus vesicular glutamate transporter 2-expressing (VGlut2) neurons that are suppressed during food intake and contribute to the disinhibition of VTA DA neurons, thus enhancing DA output to the NAc during reward consumption. In another key finding, Wang et al. [30] demonstrated that activating excitatory projections from the dorsal raphe nucleus (DRN) to VTA DA neurons in mice with neuropathic pain alleviated mechanical allodynia and reversed anhedonia-like behaviors. This intervention increased DA release in the medial part of the NAcSh through DA D1 and D2 receptor mechanisms, suggesting that DRN–VTA circuits are crucial regulators of pain-induced motivational deficits.

Recent evidence implicates the lateral habenula (LHb) in cognitive functions, primarily through cholinergic modulation [69]. Beyond its cognitive role, the LHb serves as a major afferent structure in DAergic regions, notably influencing DAergic tone indirectly [70]. The main output target of the LHb is the VTA, with which it maintains reciprocal connectivity [71,72]. According to the prevailing hypothesis on pain-related cognitive and mood disturbances, hyperactivity within the LHb leads to suppressed DA signaling in the VTA, thereby contributing to the cognitive and affective dysfunctions observed in chronic pain conditions [73,74]. Mechanistically, this occurs through LHb glutamatergic projections to VTA GABAergic interneurons, resulting in inhibitory control over DA neuron activity and modulation of reward-related signaling [75]. VTA DA neurons project widely to the cortical and subcortical areas via the mesocortical and mesolimbic pathways, subsequently mediating critical processes such as cognitive flexibility, motivational behavior, and reward-valence encoding [76,77]. Furthermore, the LHb itself receives modulatory feedback from DAergic structures, integrating both positive and negative outcome signals to regulate cognitive performance under conditions of high demand [78,79]. Therefore, disruptions within the LHb–VTA circuitry impair the processing of stimulus salience and emotional valence, playing a central role in the development of cognitive alterations associated with the chronic pain phenotype [8,72]. In this context, our previous findings demonstrated that neuropathic pain induces a reorganization of intra-LHb information processing, which may underlie the decline in cognitive accuracy observed during pain states [18].

Other neuromodulatory systems are also affected by chronic pain (Table 1). Chronic inflammation, often modeled by complete Freund’s Adjuvant (CFA) injection, alters glutamatergic and GABAergic balance in regions such as the medial PFC and amygdala. For example, it has been shown that CFA-induced inflammation reduced expression of brain-derived neurotrophic factor (BDNF) in the infralimbic cortex [80]. As BDNF is essential for synaptic plasticity and emotional regulation, its downregulation impairs the integration of affective and reward-related signals. These changes are associated with increased immobility in the forced swim test and reduced exploratory behavior [81], reflecting depression-like phenotypes. Taken together, these findings highlight how chronic pain reconfigures key components of the reward system through alterations in neurotransmitter levels, neural excitability, and synaptic plasticity. Targeting these circuits through pharmacological, neuromodulatory, or gene-based interventions offers potential avenues for reversing the affective-motivational deficits that characterize chronic pain.

## 3. Temporal Dynamics of Hedonic System Dysregulation in Chronic Pain

Understanding the temporal progression of reward system dysregulation in chronic pain is critical for identifying therapeutic windows and preventing long-term affective deficits. Preclinical studies indicate that disruptions within the mesolimbic reward circuit emerge rapidly after injury. As mentioned before, de la Puente et al. [13] reported reduced DA levels and elevated NE in the NAc within three days after PSNL, correlating with decreased palatable food intake and reduced mesolimbic activation. Similarly, Abdul et al. [87] observed impaired NAc DA release within one week of chronic constriction injury (CCI), disrupting reward consumption dynamics. These early neurochemical changes may vary between neuropathic and inflammatory pain models, with inflammatory models often exhibiting a faster onset due to more robust immune responses [88]. Optogenetic studies have further clarified the temporal dynamics underlying these processes. Markovic et al. [32] demonstrated that inhibition of VTA DA neurons shortly after CFA injection markedly impaired reward-seeking behavior, with only partial recovery following subsequent restoration of DA activity. These results suggest the existence of a critical early window for therapeutic intervention. Collectively, the findings indicate that DAergic dysfunction initiates a cascade of neurochemical and behavioral disturbances, progressively exacerbated by neuroinflammatory mechanisms that include microglial activation and cytokine release [88]. Notably, female rodents appear to develop faster or more pronounced reward deficits, potentially mediated by estrogen-dependent neuroimmune interactions [28,86].

Inflammation further disrupts monoaminergic signaling critical for reward processing. Peripheral and central inflammatory cytokines reduce precursor availability for DA and 5-HT synthesis, impair synaptic release, and enhance reuptake, ultimately lowering extracellular neurotransmitter levels [89,90]. In particular, pro-inflammatory cytokines such as TNF-α and IL-1β limit DA synthesis by decreasing tetrahydrobiopterin availability, while simultaneously increasing DA transporter activity [91]. Furthermore, astrocytes respond to these pro-inflammatory cytokines and can disrupt glial function through several mechanisms. Apart from secreting their own mediators, such as transforming growth factor beta (TGF-β) to modulate calcium signaling and regulate microglial activation [92], they can also play a role in maintaining DA dysfunction through the renin-angiotensin system (RAS). Though a direct link between neuropathic pain and RAS has not been formally established, it has been suggested that neuroinflammation could be partially driven by glial RAS, as the case has been observed in Parkinson’s disease [93,94]. These alterations, detectable through microdialysis or PET imaging, may serve as early biomarkers of affective dysfunction in chronic pain, and may help explain some forms of treatment-resistant depression and anhedonia. Early interventions targeting chronic glial activation and pro-inflammatory environments show therapeutic potential. DA agonists enhance D2/D3 receptor signaling and mitigate motivational deficits [95]. Glial inhibitors, such as minocycline, attenuate neuroinflammation and preserve DAergic function, conferring neuroprotective effects [88,96]. Moreover, behavioral strategies, including environmental enrichment and exercise, modulate DA signaling and reduce inflammatory load [97,98,99]. Targeting these early biomarkers within the first week post-injury may prevent the increased DA suppression and development of anhedonia and related mood disturbances.

Recent longitudinal and translational studies further describe how these neurochemical disruptions evolve into persistent reward deficits. For example, inflammatory processes are linked to decreased striatal D2 receptor availability in humans, supporting the hypothesis that systemic inflammation contributes to DAergic downregulation [100]. At the cellular level, recent single-cell and single-nucleus transcriptomic analyses reveal profound glial metabolic–immune rewiring and disrupted neuron–glia signaling across pain models, including neuropathic and migraine paradigms [101,102]. These studies highlight microglial and astrocytic activation as central drivers of neuroinflammatory cascades that interfere with mesolimbic DA regulation. Moreover, dynorphin-mediated KOR activation within the mesolimbic circuitry contributes to tonic suppression of DAergic firing and sustained aversive affective states, whereas KOR antagonism restores reward behavior in chronic pain models [103]. Collectively, these molecular and imaging findings reveal that early DAergic suppression transitions into stable maladaptive neuroimmune and neurochemical reorganization, underscoring the importance of timely, multimodal interventions to prevent the consolidation of hedonic deficits in chronic pain.

## 4. Sex Differences in Pain-Induced Hedonic Dysregulation

Preclinical studies consistently demonstrate that female rodents exhibit heightened pain sensitivity and greater affective distress than males, especially in models of neuropathic and inflammatory pain [27,104]. These sex differences are partly driven by fluctuations in gonadal hormones, particularly estrogens, which modulate both nociceptive thresholds and affective processing. In developmental stages, estrogens have been shown to increase spinal horn neuron excitability [105] and contribute to epigenetic modifications [106], which determine neural and immune system predisposition regarding pain sensitivity. Furthermore, estrogen can modulate the response of these systems through activation of specific pathways. For example, estrogen levels peak during the proestrus phase of the estrous cycle, coinciding with increased pain sensitivity and altered stress reactivity, while lower estrogen levels in the estrus phase may reduce this sensitivity [107]. Estrogens influence central pain processing through multiple mechanisms that increase excitability of sensory neurons, including modifications in vanilloid receptor sensitivity, nerve growth factor (NGF) signaling, and modulation of opioidergic and glutamatergic signaling, as well as enhance astrocytic and microglial activation through Toll-like receptors (TLRs) and chemokine receptors [108,109]. The latter is more pronounced in females and contributes to heightened pain perception [110]. This behavioral phenotype is accompanied by reduced DAergic activity within the VTA, indicating that chronic pain may more profoundly impair the motivational dimension of behavior in females. Correspondingly, DA D2 receptor expression and phasic DA release in the NAc are diminished to a greater extent in females under stress-related conditions [111,112,113], providing a neurochemical basis for these sex-specific vulnerabilities. Further evidence supports differential DAergic regulation between sexes. Towers et al. [114] reported that males, but not females, develop tolerance to NAc D2 receptor antagonism with the progression of an addiction-like phenotype, highlighting sex-dependent adaptations in reward circuitry. Moreover, estrogens play a well-established role in modulating DAergic signaling within the VTA-NAc pathway, shaping both reward valuation and motivational drive [115,116,117]. The estrogen hormonal modulation of the DA system underlies female-prone vulnerability to alterations in reward processing during chronic pain, increasing their susceptibility to reward-related deficits. For example, it has been shown that estrogen enhances DA transmission and increases the sensitivity of D1 and D2 receptors [41], suggesting that hormonal modulation may render females more susceptible to DA-related disruptions under stress or chronic pain. Other experimental studies also demonstrate that estrogen enhances DAergic signaling within the mesolimbic pathway, which may contribute to the heightened sensitivity of females to changes in reward-seeking behavior under chronic pain conditions [118]. Estrogen has also been shown to modulate CB1 endocannabinoid receptor activity [119], which further influences reward and affective processing under painful conditions.

Another important factor is the hypothalamic-pituitary-adrenal (HPA) axis, which regulates the stress response. Females tend to exhibit a more pronounced corticosterone response to both physical and emotional stressors [120], and more robust activation of the HPA axis in response to stress, and this elevated stress reactivity has been shown to disrupt DA homeostasis in mesolimbic regions [121,122]. As the stress response is known to be more pronounced in females, this could further exacerbate the impact of chronic pain on hedonic processing. This increased stress reactivity may contribute to the detrimental effects of chronic pain on motivation and reward-seeking behavior, creating a feedback loop of emotional distress and anhedonia. It has been shown that female rats are also less tolerant of scenarios where the higher-risk options offer larger potential outcomes compared to males [118]. This inherent risk-prone profile alone explains the increased preference for high-risk options observed in male rats with inflammatory pain [25]. Chronic pain amplifies this dysregulation, contributing to a vicious cycle of emotional distress, motivational impairment, and blunted reward sensitivity. Therefore, these sex-specific differences in pain sensitivity, stress reactivity, and reward processing underscore the critical importance of incorporating both male and female subjects into preclinical research.

Collectively, these findings underscore the necessity of incorporating both sexes in chronic pain research to better understand the sex-specific neurobiological substrates underlying pain-induced anhedonia. Although most of the research cited performed experiments using only females or both sexes for comparative analysis, estrous cycle phase control in most of them is implicit due to age and same-sex grouping, with only a handful describing in detail how they assessed for estrous phase. Given that chronic pain and associated affective disorders disproportionately affect females, elucidating the neurobiological mechanisms and hormonal contributions underlying pain-induced reward dysfunction is essential. Recognizing these sex differences also has critical translational implications. For example, pharmacological interventions targeting selective estrogen receptors or DAergic pathways may be particularly beneficial for female patients to address sex-specific hedonic deficits, whereas treatments addressing distinct neurochemical mechanisms in males could yield complementary therapeutic strategies. Targeting sex-dependent neuroimmune interactions and hormonal modulation provides promising avenues for personalized therapeutic strategies in chronic pain management [123]. The interaction between hormonal fluctuations and the neural circuits involved in pain processing is therefore critical for understanding sex differences in chronic pain.

## 5. Chronic Pain and Maladaptive Reward-Seeking

Chronic pain profoundly alters reward circuitry, diminishing the salience of natural rewards while increasing susceptibility to pharmacological reinforcers such as opioids, thereby elevating the risk of opioid use disorder [124]. This shift is largely driven by negative reinforcement, wherein opioids alleviate pain and emotional distress rather than induce euphoria. Central to this process is the KOR system: activation of KOR by dynorphin suppresses DA release in the NAc, causing dysphoria and negative affect [29,125]. Chronic pain also enhances KOR activity and further suppresses DAergic signaling [28,126]. These effects appear to be sex-dependent, with females exhibiting greater KOR-mediated negative affect likely due to hormonal modulation of the dynorphin-KOR axis [86]. Lorente et al. [86] demonstrated that inflammatory pain induces sex- and time-dependent alterations in this affective state, as females showed reduced corticosterone levels alongside changes in dynorphin, KOR, and corticotropin-releasing factor expression (CRF). Notably, KOR blockade within the NAc prevents pain-induced affective disturbances in females [86]. These findings highlight the complex interplay between opioid signaling, neuroimmune activation, and stress-related mechanisms in chronic pain. Chronic pain also drives neuroplastic remodeling within the mesolimbic pathway, particularly in the VTA and NAc [86,127]. Similar adaptations are observed in neuropathic pain models such as the PSNL, reinforcing the critical contribution of these circuits to pain-induced motivational and affective dysfunction [13]. Collectively, these neuroadaptations reduce the reinforcing properties of both natural and opioid rewards, contributing to tolerance and the escalation of opioid intake required for relief. Moreover, chronic pain enhances cue reactivity, rendering drug-associated cues potent triggers for craving and relapse [128]. Adding to this, chronic pain disrupts opioid signaling and neuroimmune interactions within the mesocorticolimbic system. In inflammatory pain models, activation of MOR in the NAc promotes the release of pro-inflammatory cytokine release (e. g. IL-1α, IL-1β, IL-6), whereas MOR blockade attenuates microglial activation across key regions, including the VTA, PFC, NAc, and amygdala [129,130]. In this regard, Thompson et al. [131] demonstrate that long-standing neuropathic injury alters the neurochemical landscape of hedonic circuitry by markedly decreasing MOR availability in structures such as the NAc and PFC. This receptor loss correlates with reduced sucrose preference and other signs of anhedonia, indicating that chronic pain reshapes reward pathways in ways that blunt affective responsiveness.

Beyond the core mesolimbic reward circuitry, several other brain regions contribute to hedonic dysregulation in chronic pain. The insular cortex plays a critical role in integrating interoceptive, affective, and cognitive components of both pain and reward. In chronic pain states, altered insular activity and disrupted connectivity with prefrontal and limbic structures destabilize the pain-reward balance, contributing to anhedonia [132,133]. The dorsal striatum, essential for action selection and habit formation, exhibits reduced activation during reward anticipation in chronic pain patients, potentially promoting a shift from goal-directed to habitual behaviors [134,135]. Similarly, hyperactivity of the amygdala, a central hub for emotional learning, amplifies negative affect and diminishes hedonic capacity [136,137]. The VP, a major NAc output that encodes hedonic value, also shows functional impairment in chronic pain, leading to blunted pleasure responses and reduced motivation [138,139]. The hypothalamus, which coordinates both pain and reward responses, undergoes significant functional alterations under chronic pain conditions, particularly within the lateral hypothalamus and paraventricular nucleus. These changes disrupt interactions between reward and stress systems, further contributing to affective and motivational deficits [140,141]. Although opioids remain essential for the management of severe pain, their potential to exacerbate reward circuit dysfunction demands user caution. Non-opioid analgesics, cognitive-behavioral therapy, and interventions aimed at restoring natural reward processing may help mitigate the risk of opioid use disorder. Importantly, KOR antagonists have shown promise in preclinical models by restoring DAergic function and alleviating negative affect, highlighting their potential as mechanism-based therapies for chronic pain-related reward dysregulation [29,126].

## 6. Behavioral Testing of Hedonic Processing in Chronic Pain

The sucrose preference test (SPT) is a widely used paradigm for assessing anhedonia, in which rodents’ preference for a sweet solution over water serves as a proxy for hedonic capacity [24]. A reduction in sucrose consumption indicates diminished pleasure, a hallmark of depressive-like states. Using the CCI model, Dellarole et al. [83] reported significant declines in sucrose preference without changes in total fluid intake, suggesting a specific hedonic deficit rather than altered thirst or fluid regulation. Similarly, Wang et al. [142] observed a persistent reduction in sucrose preference in rats subjected to the SNI model, which was accompanied by depression-like behaviors. Importantly, therapeutic interventions, including antidepressants, DAergic agents, and optogenetic VTA stimulation, restore sucrose preference, supporting SPT’s validity as a measure of pain-induced anhedonia [126,143]. Nonetheless, the SPT’s sensitivity to factors such as fluid regulation, metabolic state, and experimental conditions highlights the need to interpret results cautiously and to employ complementary assays [24].

Taste reactivity (TR) tests evaluate hedonic responses by quantifying orofacial reactions (e.g., tongue protrusions, gaping) to intraoral delivery of palatable or aversive stimuli, thereby isolating hedonic processing from motivational or motor influences [144,145]. Okun et al. [84] reported no changes in positive orofacial responses to sucrose in rats with spinal nerve ligation or CFA-induced inflammation, suggesting that core hedonic processing remains intact under chronic pain conditions. In contrast, the same animals exhibited reduced lever pressing for sucrose in operant tasks, reflecting impaired motivation. This dissociation implies that brainstem-mediated hedonic reactions may be relatively resilient to chronic pain, whereas forebrain-dependent processes such as motivation are more susceptible. Although TR tests provide an objective measure of hedonic capacity, they may overlook subtle or context-dependent changes and are most informative when combined with complementary behavioral paradigms.

Operant conditioning tasks, typically employing fixed-ratio or progressive-ratio schedules, are widely used to assess motivational drive. Progressive-ratio (PR) paradigms, in which the effort required to obtain rewards increases incrementally, are particularly sensitive to the motivational deficits induced by chronic pain. Schwartz et al. [82] demonstrated reduced PR breakpoints in both neuropathic and inflammatory pain models, indicating decreased motivation that could not be explained by physical limitations. These deficits are linked to neuroinflammatory changes and reduced DA release in the NAc [146], as well as impairments in PFC-mediated attention and executive function [147,148].

Negative affect induced by pain increases perceived effort costs, which further inhibits reward pursuit. Despite widespread evidence that chronic pain disrupts reward processing, induces anhedonia, and alters DAergic signaling, Okun et al. [84] report that neuropathic pain induced by L5/L6 spinal nerve ligation does not alter either hedonic or motivational responses to food reward in rats. Using facial reactivity scoring and operant PR, the authors found no difference between neuropathic and sham rats in “liking” or “wanting” behaviors, even up to 120 days post-injury. This finding contrasts with prior reports of diminished reward responsiveness and motivational deficits in chronic pain studies [82]. The results are particularly contradictory given robust evidence that neuropathic pain induces DAergic and affective circuit alterations that would be expected to blunt hedonic and motivational drive. This paradox can be interpreted as possibly reflecting adaptive or compensatory mechanisms that preserve food-related reward behaviors despite persistent pain, yet it still does not fully explain how observed long-term alterations in neurotransmitter, cognitive and affective systems associated with chronic pain are integrated into these findings, and what this entails in terms of behavioral manifestations to different types of rewards or rewarding events. Therefore, emerging approaches, such as real-time place preference, may provide additional insight into the dynamic aspects of reward-seeking behavior in chronic pain [14].

Integrating operant tasks with sucrose preference and taste reactivity assays offers a multidimensional evaluation of how chronic pain disrupts both hedonic experience and motivational processes. To provide an integrated view of the methods discussed above, Table 2 outlines the main behavioral tests employed to evaluate hedonic capacity and motivation, together with the domains they assess and key limitations.

## 7. Conclusions and Future Directions

The present study underscores the profound influence of chronic pain on hedonic processing, demonstrating that persistent nociceptive input extends beyond somatosensory disruption to affect motivational and affective dimensions of reward. These findings align with growing evidence that chronic pain alters mesocorticolimbic circuitry, particularly the DA and opioid systems, which are essential for encoding reward salience and pleasure. The observed impairments in reward anticipation and reduced responsiveness to natural reinforcers support preclinical models showing DAergic hypofunction and neuroinflammatory changes in key regions such as the NAc and VTA. Importantly, the data reviewed highlight the temporal complexity of these changes, suggesting that neurochemical and behavioral impairments may emerge on distinct timelines, with early DAergic disruptions preceding the onset of observable anhedonic behavior. This distinction reinforces the need for early identification of reward-related dysfunction in chronic pain patients, as it may signal vulnerability to affective comorbidities such as depression or substance misuse. Moreover, the findings of sex-specific differences in stress and reward responses emphasize the necessity of incorporating sex as a biological variable in both clinical and preclinical pain research. Together, these insights suggest that targeting hedonic deficits, through pharmacological, behavioral, or neuromodulatory strategies, may be crucial for comprehensive chronic pain treatment, particularly in improving emotional well-being and reducing the risk of maladaptive coping strategies such as opioid misuse.

The maladaptive shift in reward processing caused by chronic pain has significant implications for treatment strategies. While opioids remain a cornerstone in managing severe pain, their role in altering the reward system and increasing the risk of addiction cannot be ignored. These findings highlight the necessity of addressing the broader neurobiological and psychological factors in chronic pain management. Alternative therapies, such as non-opioid analgesics, cognitive-behavioral therapies, and behavioral interventions aimed at restoring natural reward processes, may help reduce reliance on opioids and promote healthier coping mechanisms for those living with chronic pain. Additionally, understanding how chronic pain reshapes the reward hierarchy could improve addiction risk assessment, potentially guiding clinicians to identify individuals who are at a higher risk of opioid misuse based on their altered reward sensitivities. Future research should explore the specific molecular and circuit-based mechanisms by which chronic pain induces these shifts in the reward system. For instance, the role of the opioid receptor system and its interaction with DA and 5-HT systems in chronic pain models warrants further investigation. Targeting these systems with selective pharmacological agents or behavioral interventions could provide novel therapeutic strategies for addressing both the pain and the reward-related components of opioid use disorder in chronic pain patients.

## Figures and Tables

**Table 1:** Example of preclinical studies involving cortical and subcortical areas during the encoding of hedonic and motivational associations under chronic pain.

Ref.	Pain Model	Brain Regions	Methodology	Key Findings	Implications for Hedonic/ Motivational Processing
de la Puente et al., (2022) [13]	PSNL (mice)	NAc	Microdialysis, HPLC, SPT, operant conditioning, σ1r blockade	Decreased sucrose preference and operant responses to food rewards, indicating anhedonia. Reduced extracellular DA and increased 5-HT in NAc; blunted DAergic and 5-HTergic responses to palatable stimuli; σ1r blockade restored DA and 5-HT levels.	CP induces anhedonia via disrupted DAergic and serotonergic signaling in NAc; σ1r modulates reward signaling.
Markovic et al., (2021) [32]	CFA (mice and rat)	VTA, NAc	Optogenetic and chemogenetic stimulation, operant conditioning	Reduced operant performance for sucrose rewards in pain model; restored by VTA DA stimulation. Inhibition of VTA DA neurons post-CFA impaired reward-seeking; optogenetic VTA DA stimulation in SNI mice restored operant performance for sucrose rewards.	VTA DA hypofunction is a causal factor in pain-induced anhedonia; motivational deficits are reversible with targeted stimulation.
Wang et al., (2023) [30]	SNI (mice)	DRN, VTA, Medial NAc	Optogenetic activation, behavioral assays (mechanical allodynia, SPT)	Reduced mechanical allodynia and restored sucrose preference in neuropathic pain models. Activation of DRN-VTA excitatory projections alleviated mechanical allodynia and reversed anhedonia-like behaviors; increased DA release in medial NAc via D1/D2 receptors.	DRN-VTA circuit regulates pain-induced motivational deficits; enhanced DA release in medial NAc restores hedonic tone.
Schwartz et al., (2014) [82]	SNI, CFA (mice)	NAc	PR operant tasks, immunohisto-chemistry	Decreased motivation for rewards, not explained by physical limitations. Reduced PR breakpoints in operant tasks; linked to neuroinflammatory changes and reduced DA release in NAc.	CP impairs motivation via NAc neuroinflammation and DAergic hypofunction.
Cardoso-Cruz et al., (2022) [17]	SNI (rat)	PL, NAcC	Electrophysiology, WM tasks	Altered prefrontal-striatal theta-band oscillatory dynamics; WM deficits in neuropathic pain rats.	Disrupted prefrontal-striatal connectivity contributes to cognitive and motivational impairments in CP.
Cardoso-Cruz et al., (2024) [18]	SNI (rat)	LHb	Electrophysiology, spatial memory tasks	Reduced cognitive accuracy in spatial memory tasks. Reorganization of intra-LHb connectivity; impaired spatial memory encoding linked to pain-related cognitive deficits.	LHb hyperactivity suppresses DA signaling, contributing to cognitive and affective dysfunctions in CP.
Dellarole et al., (2014) [83]	CCI (mice)	Hippocampus	SPT, TNFR1 signaling analysis	Reduced sucrose preference; depressive-like behaviors linked to TNFR1 signaling and impaired hippocampal neurogenesis.	Hippocampal plasticity changes contribute to anhedonia and depressive phenotypes in CP.
Okun et al., (2016) [84]	SNL, CFA (rat)	Not applicable	TR test, operant conditioning	Intact hedonic responses but impaired motivational drive in operant tasks. No change in positive orofacial responses to sucrose in TR tests; reduced lever pressing in operant tasks.	Dissociation between preserved brainstem-mediated hedonic responses and forebrain-dependent motivational deficits in CP.
Taylor et al., (2015) [85]	PNI (mice)	NAc, VTA	Microglial analysis, DA release measurement	Reduced reward consumption and motivation in CP. Microglia-mediated disruption of mesolimbic reward circuitry; reduced DA release in NAc linked to motivational deficits.	Neuroinflammation in NAc and VTA impairs reward processing, contributing to motivational deficits.
Lorente et al., (2024) [86]	CFA (rat)	NAcSh	Pharmacological modulation, behavioral assays	Reduced sucrose preference and increased negative affect, more pronounced in females. Sex- and time-dependent negative affect; KOR blockade in NAcSh prevented pain-induced affective disturbances in females.	KOR-mediated negative affect in NAcSh drives pain-induced hedonic dysregulation, with sex-specific effects.
Abdul et al., (2022) [87]	CCI (mice)	VTA, NAcSh	Optogenetic modulation, Microdialysis, behavioral assays	Reduced palatable food intake and reward-seeking behavior.Impaired NAc DA release within one week after CCI; disrupted reward consumption dynamics.	Early DAergic impairments in NAc contribute to motivational deficits in CP.

Abbreviations: 5-HT, serotonin; 5-HTergic, serotonergic; σ1r, sigma-1 receptor; CCI, chronic constriction injury; CFA, Complete Freund Adjuvant; CP, chronic pain; DA, dopamine; DAergic, dopaminergic; DRN, dorsal raphe nucleus; HPLC, high-performance liquid chromatography; KOR, κ-opioid receptor; LHb, lateral habenula; NAc, nucleus accumbens; NAcC, nucleus accumbens core; medial NAc, medial nucleus accumbens; NAcSh, nucleus accumbens shell; PL, prelimbic cortex; PNI, partial nerve injury; PR, progressive-ratio operant task; PSNL, partial sciatic nerve ligation; SNI, spared nerve injury; SPT, sucrose preference test; TNFR1, tumor necrosis factor receptor 1; TR, taste reactivity test; VTA, ventral tegmental area; WM, working memory.

**Table 2:** Overview of behavioral preclinical tests assessing reward processing.

Behavioral Test	Description	Assessment	Limitations
Sucrose preference test (SPT)	Animal chooses between two solutions, one of them containing sucrose (% of liquid consumed)	Reward sensitivity	Experimental protocols: inconsistency in test duration and preceding conditions of water and food deprivation
Taste reactivity test (TR)	Intraoral delivery of palatable or aversive stimuli (assessment of orofacial responses)	Responses to gustatory stimuli	Difficulty to evaluate and interpret affective reactions by experimenters
Operant conditioning fixed-ratio (FR)	Animal performs a certain number of responses to receive reward	Motivation	Potential for devaluation of activity due to certainty
Operant conditioning progressive-ratio (PR)	Animal performs an incremental number of responses to receive reward	Motivation	Difficulty in separating hedonic value (liking) from motivation (wanting)
Conditioned place preference (CPP)	Animal chooses between two chambers with distinct cues and substances/protocols	Motivation	Possibility of confounding effects with exploration; Difficulty in separating hedonic value (liking) from motivation (wanting)
Intracranial self-stimulation	Animal delivers brief electrical pulses into his own brain	Motivation	Requires invasive procedures; Difficulty in separating hedonic value (liking) from motivation (wanting)

## Data Availability

No new data were created or analyzed in this study.

## Abbreviations

The following abbreviations are used in this manuscript:

5-HT	Serotonin
5-HTergic	Serotoninergic
ACC	Anterior cingulate cortex
BDNF	Brain-derived neurotrophic factor
CCI	Chronic constriction injury
CFA	Complete Freund’s adjuvant
CRF	Corticotropin-releasing factor
CP	Chronic pain
DA	Dopamine
DAergic	Dopaminergic
DRN	Dorsal raphe nucleus
GABA	Gamma-aminobutyric acid
HPA	Hypothalamic-pituitary-adrenal
HPLC	High-performance liquid chromatography
IL-1α	Interleukin 1 alfa
IL-1β	Interleukin 1 beta
IL-6	Interleukin 6
KOR	κ-opioid receptor
LHb	Lateral habenula
MEF2C	Myocyte enhancer factor 2C
MOR	µ-opioid receptor
NAc	Nucleus accumbens
NAcC	Nucleus accumbens core area
Medial NAc	Nucleus accumbens medial shell area
NAcSh	Nucleus accumbens shell area
NE	Norepinephrine
NGF	Nerve growth factor
NP	Neuropathic pain
OFC	Orbitofrontal cortex
PFC	Prefrontal cortex
PL	Prelimbic cortex
PNI	Peripheral nerve injury
PR	Progressive-ratio operant task
PSNL	Partial sciatic nerve ligation
RAS	Renin-angiotensin systrem
σ1r	Sigma-1 receptor
SNI	Spared nerve injury
SNL	Spinal nerve ligation
SPT	Sucrose preference test
TGF-β	Transforming growth factor beta
TLRs	Toll-like receptors
TNF-α	Tumor necrosis factor alfa
TNFR1	Tumor necrosis factor receptor 1
TR	Taste reactivity test
WM	Working memory
VGlut2	Vesicular glutamate transporter 2
VP	Ventral pallidum
VTA	Ventral tegmental area
